# Differential effects of taurine treatment and taurine deficiency on the outcome of renal ischemia reperfusion injury

**DOI:** 10.1186/1423-0127-17-S1-S32

**Published:** 2010-08-24

**Authors:** Mahmood S Mozaffari, Rafik Abdelsayed, Champa Patel, Hereward Wimborne, Jun Yao Liu, Stephen W Schaffer

**Affiliations:** 1 Department of Oral Biology, Medical College of Georgia School of Dentistry, Augusta, Georgia 30912, USA; 2 Department of Oral Health and Diagnostic Sciences, Medical College of Georgia School of Dentistry, Augusta, Georgia 30912, USA; 3 Department of Pharmacology, University of South Alabama Mobile, Alabama 36688, USA

## Abstract

Taurine possesses membrane stabilization, osmoregulatory and antioxidant properties, aspects of relevance to ischemic injury.  We tested the hypothesis that body taurine status is a determinant of renal ischemic injury.  Accordingly, renal function and structure were examined in control (C), taurine-treated (TT) and taurine deficient (TD) rats that were subjected to bilateral renal ischemia (60 min) followed by reperfusion (IR); sham operated rats served as controls.  Baseline urine osmolality was greater in the TD group than in the control and the TT groups, an effect associated with increased renal aquaporin 2 level.  The IR insult reduced urine osmolality (i.e., day-1 post insult); the TD/IR group displayed a more marked recovery in urine osmolality by day-6 post insult than the other two groups.  Fluid and sodium excretions were lower in the TD/IR group, suggesting propensity to retention.  Histopathological examination revealed the presence of tubular necrotic foci in the C/IR group than sham controls.  While renal architecture of the TD/IR group showed features resembling sham controls, the TT/IR group showed dilated tubules, which lacked immunostaining for aquaporin 2, but not 1, suggestive of proximal tubule origin.  Finally, assessment of cell proliferation and apoptosis revealed lower proliferation but higher apoptotic foci in the TT/IR group than other IR groups.  Collectively, the results indicate that body taurine status is a major determinant of renal IR injury.

## Introduction

Acute renal failure is a potentially reversible condition with the hallmark feature of impaired ability of the kidney to eliminate waste products and maintain fluid and electrolyte balance.  It describes a syndrome of disorders that are classified into three categories: pre-renal (e.g., due to a reduction in glomerular perfusion), post-renal (e.g., due to obstruction to urine flow) and intrinsic (e.g., due to tubular necrosis).  Importantly, intrinsic acute tubular necrosis is most commonly attributable to ischemic injury which accounts for about 50% of the cases of acute renal failure [1,2].  Examples of clinical conditions associated with renal ischemia reperfusion (IR) injury include renal transplantation, partial nephrectomy and repair of some forms of abdominal aneurysms.  Indeed, despite great strides, renal IR injury associated with transplantation contributes importantly to delayed graft function, delayed graft rejection, acute rejection and chronic allograft nephropathy [3-5].

	Acute renal IR injury is a highly coordinated process that is mediated by components of both the innate and adaptive arms of immunity, which determine both the early phase and long-term functional outcome [6-11].  The ischemic insult increases endothelium permeability and expression of adhesion molecules that are crucial for recruitment and infiltration of inflammatory cells into the post-ischemic region.  Further, activation of transcription factors (e.g., nuclear factor κB) causes upregulation of inflammatory genes [6].  Upon reperfusion, the ischemic-primed endothelial cells are prone to leukocyte migration and platelet adhesion, which result in further enhancement of endothelial cell permeability and cell activation.  In turn, leukocytes serve as a source of reactive oxygen species and a variety of cytokines, which further exacerbate the inflammatory process.  These processes, coupled with IR injury-induced loss of cellular energy and consequent derangement of ionic homeostasis ultimately lead to cell death [6-10].  Proximal tubular cells, a prime target of IR injury, swell, lose brush borders and develop cytoskeletal abnormalities, including abnormal localization of cell membrane components (e.g., translocation of the Na^+^-K^+^-ATPase from the basolateral sites to the cytoplasm/apical sites of the tubular cells) [7-9,11].  Functional consequences include decreased tubular reabsorption of sodium, as reflected by the increase in fractional excretion of sodium [10].  Indeed, as a result of the enhanced delivery of solutes to the macula densa, the tubuloglomerular feedback mechanism is activated leading to a persistent constriction of the afferent arterioles and a consequent reduction in the glomerular filtration rate [1,7,10].   Another functional hallmark of renal IR injury is marked impairment in urinary concentrating ability, which is further evidence for the dysregulation of tubular fluid and of solute transport [10].  Histological examination of the kidney reveals tubules surrounded by flattened, denuded epithelium containing lumen filled by cell debris; peritubular capillaries are congested and display extensive inflammatory infiltrates [1,2,7].

	Interestingly, renal tubule cells possess a remarkable ability to regenerate and proliferate following an ischemic injury [1,7,9].  While some have implicated mesenchymal stem cells (resident and bone marrow-derived) in the regeneration of tubule cells [12,13], others have implicated the dedifferentiation of viable cells, which subsequently proliferate, differentiate and establish polarity, thereby restoring normal structure and function [8,11].  The return of glomerular filtration contributes to the removal of tubular debris to minimize obstruction.  A period may exist in which glomerular filtration has normalized but tubular function remains deranged.  This period underlies the polyuric phase of acute tubular necrosis associated with hypoosmotic polyuria.  Indeed, it has been suggested that severe ischemic injury results in permanent alterations in renal capillary density that not only contributes to the urinary concentrating deficit but also predisposes to the development of later complications (e.g. altered sodium homeostasis, propensity to hypertension and secondary renal disease) [2,3,10].

The marked adverse impact of IR injury on the kidney necessitates identification of effective maneuvers and therapies aimed at preventing and limiting tissue injury.  Taurine is shown to exert a number of cytoprotective effects that could potentially benefit the ischemic reperfused kidney.  These include its purported antioxidant, membrane stabilizing, cellular osmoregulatory and antiapoptotic effects [14-17].  In addition, we have shown that taurine is an important modulator/ regulator of renal function and therefore a contributor to body fluid and electrolyte homeostasis [18-21].  Thus we conjectured that body taurine status is an important determinant of the outcome of renal IR injury.  Accordingly, we have examined the impact of taurine supplementation and deficiency on the outcome of a bilateral renal IR insult in rat.

## Methods

### Preparation of experimental groups

**A) Animals:** Male Sprague-Dawley rats (8-9 weeks of age) were obtained from Harlan laboratories (Indianapolis, Indiana).  The use of animals in this study was in accordance with the NIH guide on Humane Treatment of Laboratory Animals.  Two days after arrival, the animals were randomly assigned to receive tap water (control), tap water containing 1% taurine or tap water containing 3% β-alanine for 2 weeks; the β-alanine regimen reduces renal tissue taurine content by about 50% [21].  The animals had free access to drinking fluid and food (Harlan Teklad diet 8604) throughout the study.

**B) Bilateral ischemia reperfusion injury:** Two weeks after initiation of the treatment regimens, and collection of baseline excretion data (below), each rat was subjected to one hour of bilateral renal ischemia [10,3].  Under pentobarbital anesthesia (40-45 mg/kg; i.p.), two flank incisions were made in areas corresponding to each kidney.  Ischemia was induced by application of a nontraumatic clamp to each renal pellicle for a period of 60 minutes followed by removal of the clamp to allow continued perfusion that was verified visually before closure of surgical sites.  Sham-operated control animals were subjected to the same procedure whereby each kidney was gently manipulated but was not subjected to ischemia.  The muscle layers and the skin incisions were closed with sterile silk sutures and autoclips, respectively; postoperative analgesia was provided with butorphanol (2 mg/kg; twice daily for one day).  Thus the protocol outlined above produced four experimental groups (n=5-7 animals/ group) as follows: sham control, ischemic-reperfused (IR) control (C/IR), taurine deficient (TD) ischemic-reperfused (TD/IR) and taurine-treated (TT) ischemic-reperfused (TT/IR).  Once fully awake, each animal was returned to its metabolic cage and daily urine samples collected for 6 consecutive days.

### Renal function studies

**A)** Prior to bilateral IR injury, the animals were placed individually in metabolic cages.  After a period of two days of acclimation, two consecutive 24-hr. urine samples were collected and used for determination of baseline renal functional parameters [19-21].  Following the surgical procedure to induce IR injury, and full recovery of consciousness, the animals were returned to their metabolic cages and daily urine samples collected for 6 days.

**B)** Following daily metabolic studies, the animals were implanted with femoral vessels and bladder catheters in preparation for acute renal function studies.  Accordingly, renal responses to an isotonic saline volume load (5% body weight over 30 min., i.v.; total of 90 minute post-infusion collection) were determined in pre-instrumented conscious animal.  ^3^H-Inulin was used for measurement of the glomerular filtration rate (GFR) [18-21].

### Histopathological studies

Formalin-fixed and paraffin-embedded kidney tissue blocks were cut in 4 µm sections followed by hematoxylin-eosin (H&E) staining.  Immunohistochemical staining was carried out utilizing aquaporin (AQP) 1 and AQP2 antibodies (rabbit-polyclonal antibodies to AQP1 and AQP2 (Sigma, St. Louis MO) [21,22].  Immunofluoresence staining for proliferating nuclear cell antigen (PCNA) was achieved with monoclonal mouse anti-PCNA (Dako, Glostrup, Denmark) for one hour. Cyanine conjugated horse anti-mouse (Jackson Immunoresearch, West Grove, PA) was used for fluorescent detection.  Finally, formalin-fixed, paraffin-embedded kidney tissues were processed for terminal deoxynucleotidyl transferase dUTP nick end labeling (TUNEL) assay using the ApopTag Plus Peroxidase in Situ Apoptosis Detection Kit (Millipore Temecula, CA).

### Western blot for AQP 2

Additional groups of control, TD and TT rats (n=3/group), which were not subjected to ischemic injury, were used for determination of the potential impact of body taurine status on (baseline) renal AQP2 expression [22].  Upon sacrifice, renal tissue was quickly procured and stored in liquid nitrogen.  Thereafter, renal tissue was powdered using a mortal and pestle (on dry ice).  The tissue powder was added to the isolation buffer (10mM triethanolamine, 250mM Sucrose, PH 7.6, 1 µg/ml Leupeptin, PMSF (2 mg/ml), sonicated and sodium dodecyl sulfate added to a final concentration of 1% prior to centrifugation; the supernatant was used for protein assay [22,23].  Standard protocols were used for Western blot analysis as described previously (i.e., 10% gel, electrophoretic protein transfer to nitrocellulose membrane, primary antibody to aquaporin 2, secondary antibody and detection by enhanced chemiluminescence; GE Healthcare UK, Buckinghamshire UK) [22].  The protein expression data were corrected for β-actin and expressed as percent of the control group.

### Assays and urinary protein profile

Sodium and potassium were measured by flame photometry and used to calculate excretion rates.  Urine osmolality was measured by an osmometer and urinary protein content by the Coomassie blue method (Sigma, Saint Louis, MO); urinary protein profile was determined using sodium dodecyl sulfate polyacrylamide gel electrophoresis (SDS-PAGE) as described previously [21].  The glomerular filtration rate and fractional excretions of sodium and potassium were calculated using standard clearance formulas [18-21].  For taurine measurement, renal tissue was homogenized in 2% perchloric acid (1:10 w/v) and the supernatant used to assay taurine by a colorimetric method [21].

### Statistics

All data were analyzed by the analysis of variance (ANOVA).  Variables that were measured sequentially were analyzed by repeated measure ANOVA.  Duncan’s post hoc test was used for comparison of mean values (significance of criteria of p<0.05).  Data are reported as means ± SEM.

## Results

Body weight was similar among the groups before the initiation of the bilateral IR protocol.  The TD/IR group (272 ± 4 g) displayed reduced (p<0.05) body weight about 10 days after initiation of the IR protocol compared to the sham control (317 ± 5 g), the C/IR group (318 ± 9 g) and the TT/IR group (310 ± 6 g).  Kidney weight was greater in the TT/IR group (3.7 ± 0.5 g) than in the sham control (2.7 ± 0.2 g; p<0.05), C/IR (3.0 ± 0.1 g) and the TD/IR groups (3.3 ± 0.2 g).  Kidney weight to body weight ratios (mg/g) were similar in the TD/IR (12.3 ± 0.7) and the TT/IR groups (12.3 ± 1.4) but higher (p<0.05) than either the C/IR (9.8 ± 0.4) or the sham controls (8.7 ± 0.7).  The TD/IR group displayed a 25% reduction (p<0.05) in tissue taurine content (µmoles/g wet tissue weight) compared to the other 3 groups (13.4 ± 0.4 [sham control]; 13.1 ± 0.6 [C/IR]; 10.5 ± 0.7 [TD/IR]; 13.4 ± 0.4 [TT/IR]).  It is noteworthy that inclusion of 3% β-alanine in the drinking water usually reduces renal tissue taurine content of control animals by about 50% [21].  Taken together, the data suggest a reduced efficacy of β-alanine to diminish kidney taurine content during the recovery phase of the IR insult.

**Figure 1:**
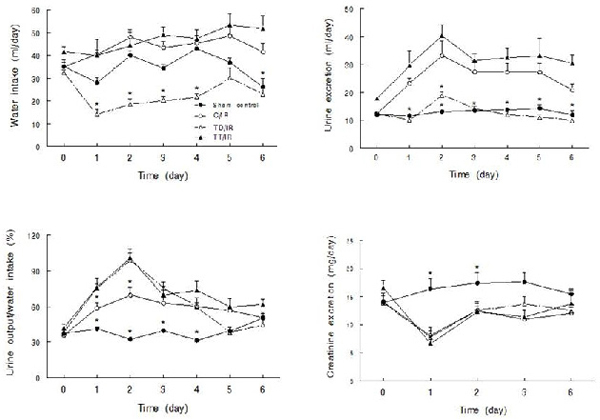
**Daily metabolic parameters of experimental groups.** Line graphs show daily water intake (A), daily urine excretion (B), the ratio of urine output to water intake (C) and daily creatinine excretion (D) prior to (day 0) and after induction of bilateral renal ischemia reperfusion (IR) injury in control (C/IR), taurine deficient (TD/IR) and taurine treated (TT/IR) as well as sham controls.  Data are means ± SEM (n=5-7 animals/group). * p< 0.05 compared to other groups at the same time point

As shown in Figure 1A, baseline (i.e., day 0) fluid intake was slightly higher (p<0.05) in the TT/IR than in the TD/IR group.  Initiation of IR was associated with greater water intake in the C/IR group.  By contrast, TD/IR showed a significant reduction in daily water intake.  Six days after initiation of IR, daily water intake was similar in the sham control and the TD/IR group, but lower (p<0.05) than those of the C/IR and the TT/IR groups.  Baseline urine excretion was higher (p<0.05) in the TT/IR group than in the other 3 groups, which all showed similar values (Figure 1B, day 0).  The initiation of IR was associated with a significant increase in daily urine excretion that peaked by day-2 followed by a modest decline towards baseline values by day-6 after initiation of IR, a pattern comparable in the control and TT groups.  By contrast, the TD/IR group showed a slight and delayed (day-2) increase in urine excretion followed by a return to the baseline value and that of the sham control.  The ratio of urine output/fluid intake was significantly higher in the 3 IR groups than in sham control, a characteristic feature of the polyuric phase of IR injury; this ratio was maximal at day-2 followed by return to baseline values by day-6 post insult (Figure 1C).  Baseline daily creatinine excretion was similar among the groups.  However, consistent with previous reports indicating a transient decrease in creatinine clearance [10], creatinine excretion was significantly reduced in the 3 IR groups but rebounded towards baseline values by day-6 post insult (Figure 1D).

**Figure 2:**
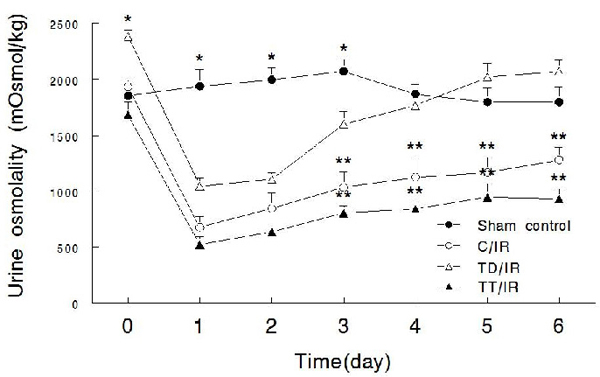
**Effects of a bilateral renal ischemia reperfusion insult on urine osmolality.** Line graphs show urine osmolality in experimental groups as described under Figure 1.  Data are means ± SEM (n=5-7 animals/group). * p< 0.05 compared to other groups at the same time point. ** p<0.05 compared to the TD/IR or the sham controls.

**Figure 3:**
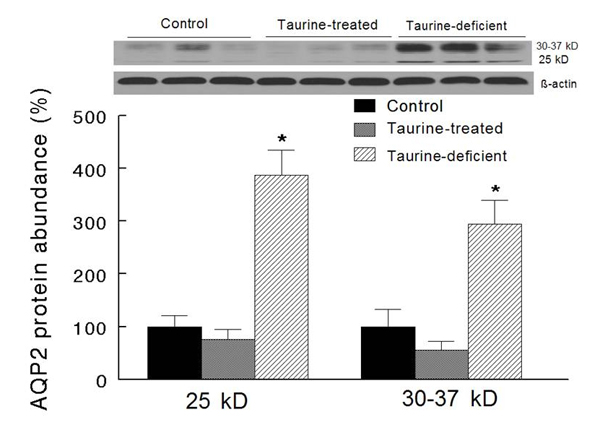
**Renal aquaporin (AQP)2 levels in taurine-treated and taurine-deficient rats.** The 25 kD and the 30-37 kD bands represent non-glycosylated and glycosylated forms of AQP2, respectively. Protein expression is expressed as percent of the control group.  Data are means ± SEM (n=3 animals/groups). * p<0.05 compared to the other two groups.

Baseline urine osmolality was significantly higher in the taurine deficient group compared to the other groups (Figure 2, day 0) [18,21].  Since renal AQP 2 is fundamental to the regulation of urine osmolality [24], we determined protein abundance of the whole kidney procured from control, taurine-deficient and taurine-treated groups that had not been subjected to the IR insult.  As shown in Figure 3, taurine-deficient kidneys showed significantly higher levels of non-glycosylated (25 kD) and glycosylated (30-37 kD) forms of AQP 2 than the control and taurine treated groups, which both showed similar AQP 2 levels.

 Initiation of IR injury was associated with a similar, marked decline in urine osmolality at day-1 post-insult in control, taurine-deficient and taurine-treated groups compared to the sham controls (Figure 2).  While the C/IR and the TT/IR groups showed a similar, partial recovery of urine osmolality, urine osmolality remained significantly lower than their respective baseline values at day-6 post insult.  By contrast, the TD/IR group displayed an accelerated recovery, which commenced on day-3; by day-6 post-insult, urine osmolality closely approached the baseline pre-ischemic-reperfusion value (Figure 2).

**Figure 4:**
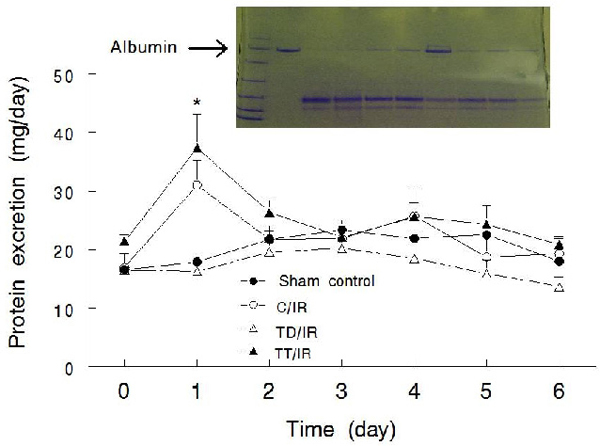
**Effects of a bilateral renal ischemia reperfusion insult on urinary protein excretion.** Line graphs show daily total protein excretion in experimental groups as described under Figure 1; data are means ± SEM (n=5-7 animals/group).  Inset shows appearance of albumin in urine of the 3 IR groups.  Lane 1 shows molecular weight standards (6.5-200kDa) while lane 2 is a sample of bovine serum albumin from comparison (arrow). Lanes 3-10 show urinary protein migration for the experimental groups as follows: 3-4 (sham control), 5-6 (C/IR), 7-8 (TD/IR) and 9-10 (TT/IR).  * p<0.05 C/IR and TT/IR compared to the other two groups at the same time point.

Daily proteinuria was similar in the groups prior to initiation of IR or the sham procedure (i.e., day 0, Figure 4).  The C/IR and TT/IR groups displayed a transient increase in proteinuria (at day 1) which returned to levels similar to the other 2 groups (e.g., days 2-4).  Nonetheless, the TD/IR group displayed a modestly lower rate of protein excretion than the TT/IR group at days 5-6 post insult (Figure 4).  Analysis of the molecular weight distribution of urinary proteins revealed the appearance of albumin in the 3 IR groups compared to the sham control group (Figure 4, day 6).

**Figure 5:**
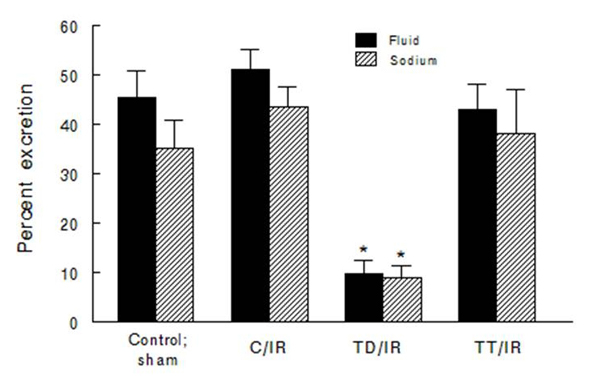
**Diuretic and natriuretic responses of experimental groups to intravenous administration of a saline volume load.** Bar graphs show saline volume-induced diuretic and natriuretic responses of experimental groups expressed as percent of the administered fluid and sodium that were excreted over 90 minutes after initiation of saline volume expansion (5% of body weight administered over 30 minutes; i.v.).  Data are means ± SEM (n=4 animals per group). *p<0.05 compared to other groups.

As shown in Figure 1B, daily urine excretion remained higher at day-6 post-insult in the C/IR and TT/IR groups than in the TD/IR and sham controls.  Similarly, six days post-insult, daily sodium excretion (mEq/day) was lower in the TD/IR (0.6 ± 0.1) than in the other three groups (1.3 ± 0.1 [sham control; p<0.05]; 1.0 ± 0.2 [C/IR]; 0.9 ± 0.2 [TT/IR]); potassium excretion (mEq/day) was numerically lower in the TD/IR (2.6 ± 0.3) and the TT/IR (3.1 ± 0.2) compared to the C/IR (3.7 ± 0.7) and sham controls (3.8 ± 0.6) by day-6 post-insult.  Thus, in order to further assess the impact of IR injury on kidney function, renal excretory responses to acute plasma volume expansion were examined in conscious animals (at day 10-11 post insult).  The TD/IR group displayed a significant reduction in saline volume-induced diuresis and natriuresis compared to the other 3 groups, all of which excreted similar percentages of the administered loads (Figure 5). The baseline GFR was similar among the groups (0.7-0.9 ml/min/g kidney weight).  Analysis of GFR and fractional excretion data indicate that increased tubular reabsorption was the primary contributor to the reduction in saline volume-induced diuresis and natriuresis in the TD/IR group (data not shown).

**Figure 6:**
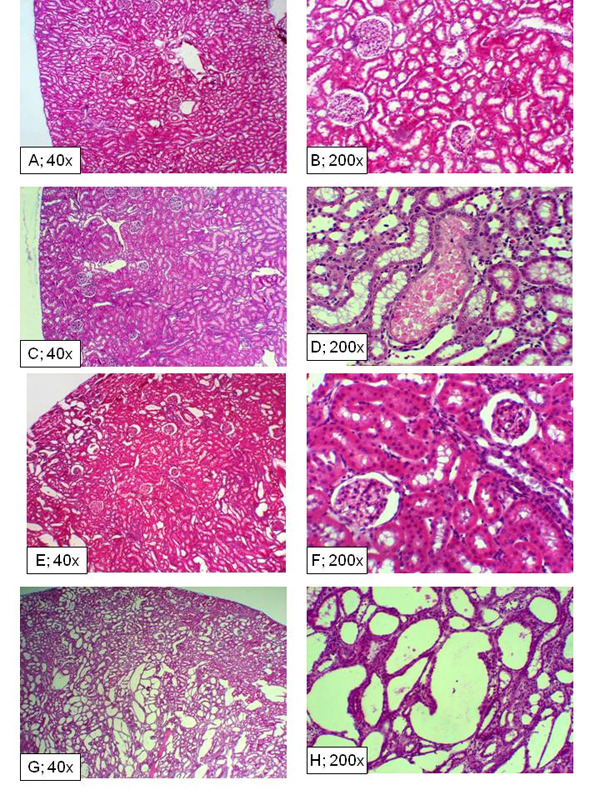
**Histopathological features of renal tissue subjected to ischemia reperfusion insult.** Panels show hematoxylin-eosin (H&E) sections of kidneys from experimental groups at day-11 post-insult: panels A and B (sham control), C and D (C/IR), E and F (TD/IR) and G and H (TT/IR).

**Figure 7:**
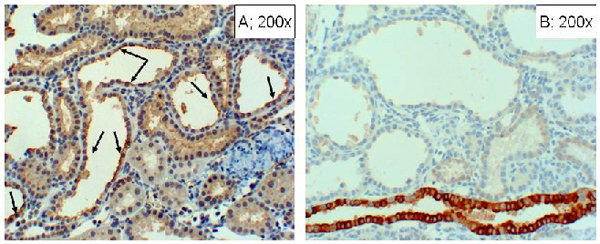
**Immunohistochemical localization of renal AQP1 and AQP2.** Panel A shows immunostaining for AQP1 antibody (arrows) while panel B shows immunostaining for AQP 2 in renal tissue of TT/IR group.

At the conclusion of the renal function studies, kidney tissues were processed for light microscopic examination.  As shown in Figure 6, the C/IR group showed persistent foci of tubular necrosis despite recovery of certain regions from the insult (panels C and D).  Interestingly, the TD/IR group (Figure 6; panels E-F) displayed features more closely resembling the sham control group (panels A and B).  In contrast to the other two IR groups, the TT/IR group exhibited a predominance of tubules displaying cystic-like features (i.e., panels G and H; Figure 6).  As shown in Figure 7, renal tubules of TT/IR group displaying cystic dilatations exhibited immunostaining for AQP1 (panel A) but not AQP2 (panel B).

**Figure 8:**
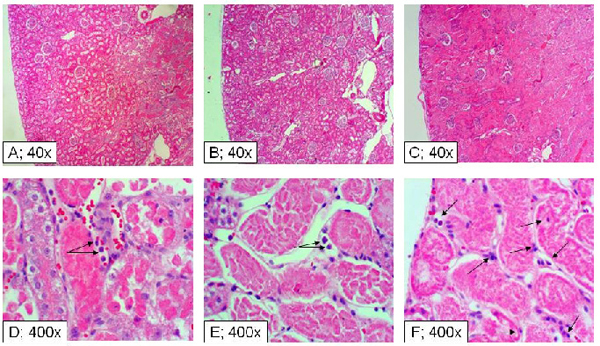
**Histopathological features of the kidney from experimental groups.** Panels show H&E sections of renal tissue from C/IR (A and D), TD/IR (B and E) and TT/IR (C and F) groups one day after induction of ischemia reperfusion insult.  Arrows point to inflammatory cell infiltrates.

In light of these observations, as well as the demonstration of a similar decline in urinary concentrating deficit in the 3 IR groups (i.e., day-1), morphological features of kidneys were explored in additional rats at day-1 post-ischemia reperfusion insult.  While the cortical region of the C/IR kidneys appeared relatively preserved, the vast majority of tubules in the medullary regions displayed sloughing, anucleated markedly eosinophilic cells (Figure 8, panels A and D).  The TD/IR group displayed morphological features resembling C/IR (i.e., apparent preservation of cortical tubules and glomeruli but marked medullary tubular necrosis; Figure 8, panels B and E).  On the other hand, the TT/IR kidneys showed diffuse cortical and medullary tubular necrosis but the glomeruli appeared structurally intact (Figure 8, panels C and F).  Interestingly, the TT/IR group showed more marked inflammatory cell infiltrates in renal tissue one day after initiation of the IR insult, compared to the TD/IR and C/IR groups, which showed moderate to mild inflammatory infiltrates (Figure 8; arrows).

**Figure 9:**
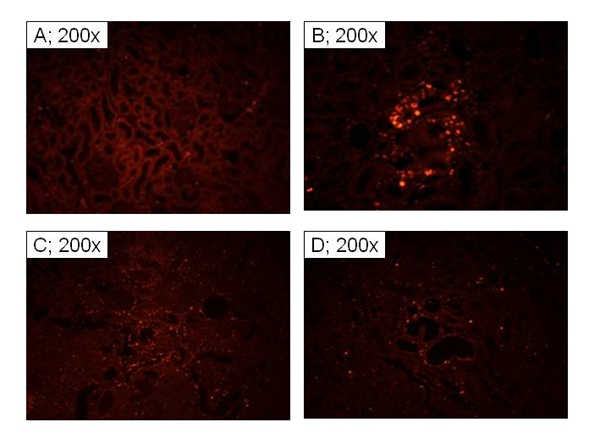
**Assessment of cell proliferation in renal tissue of experimental groups.** Panels show immunofluoresence staining for proliferating cell nuclear antigen of renal tissue from sham control (A), C/IR (B), TD/IR (C) and TT/IR (D).

**Figure 10:**
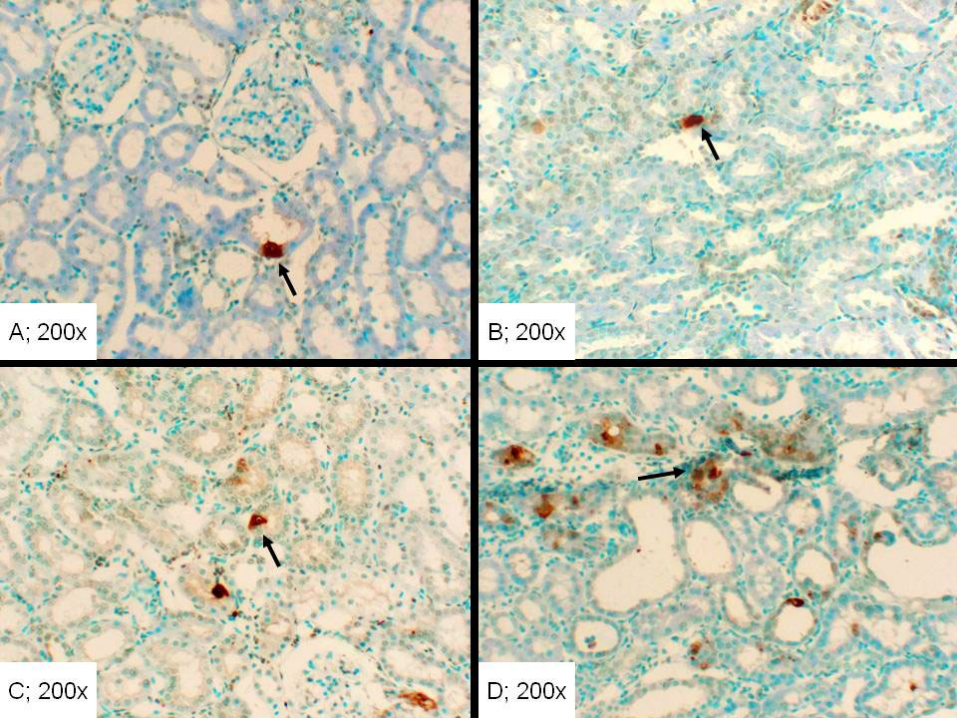
**Assessment of apoptosis in renal tissue of experimental groups.** The TUNEL assay reveals greater apoptotic foci (arrow) in the TT/IR (panel D) than the TD/IR (panel C) group; the sham control (panel A) and C/IR (panel B) showed similar, but lower, foci than the TD/IR and TT/IR groups.

Apoptosis and cell proliferation are pivotal to the recovery of the kidney from an IR insult [7-9,11].  Accordingly, we conjectured that the differential in recovery of renal architecture in the three groups relates to taurine-mediated modulation of proliferative capacity and apoptosis.  As shown in Figure 9, kidney sections from control animals showed the lowest proliferative foci (e.g., a single PCNA positive cell/tubule; Figure 9, panel A). On the other hand, tissue sections from the C/IR group showed clusters of PCNA positive cells (e.g., a range of 12-20 and an average of 16 PCNA positive cells/tubule; Figure 9, panel B).  Kidney sections from TD/IR group showed a similar high proliferative cells of varying intensity that appeared more spread throughout renal parenchyma (Figure 9, panel C). On the other hand, kidney sections from the TT/IR group showed reduced proliferative foci (e.g., a range of 4-8 and an average of 6 cells/tubule; Figure 9, panel D).  The TUNEL assay revealed foci of cellular, both nuclear and cytoplasmic, staining mainly of tubular epithelial cells and occasionally within interstitial space in all groups (Figure 10). However, the sham control and C/IR group showed the mildest reaction with only few scattered positive single cells within occasional tubules (Figure 10A-10B). The TD/IR group showed mild to moderate reaction with scattered foci of about one-to-two renal tubular cell aggregates of positive cellular reaction (Figure 10C). A more intense reaction was observed in the TT/IR group which showed several positive tubular epithelial cells within morphologically normal renal tubules juxtaposed to the areas of cystic dilation (Figure 10D).

## Discussion

The present study shows that body taurine status is a major determinant of renal IR injury.  Taurine deficiency increases both the extent and the rate of recovery of urinary concentrating ability associated with renal architecture resembling normal kidney.  By contrast, while taurine supplementation does not affect functional recovery (e.g., urinary concentrating deficit) from an IR insult, it unexpectedly causes marked dilatation of renal tubules that resemble cyst formations.  Taken together, the results show differential effects of taurine deficiency and taurine treatment on the outcome of renal IR insult.  Importantly, to our knowledge, this is the first demonstration of a marked and potentially adverse impact of taurine treatment in a pathological condition.

A number of studies have reported beneficial effects of taurine treatment on renal function and structure in chronic renal dysfunction/failure [25-28,19,20].  However, very limited information is available in relation to taurine and acute renal failure secondary to ischemic injury.  One study examined the effect of taurine on 40 minutes of bilateral renal ischemia followed by 6 hours of reperfusion [29].  Based on examination of several plasma markers and renal architecture, the authors concluded that taurine treatment reduces renal IR injury.  Nonetheless, in that study, taurine was delivered intraperitoneally during the initiation of the IR insult, making it difficult to discern its bioavailability.  A more recent study examined the impact of taurine preconditioning of the rat donor kidney; taurine preconditioning was achieved by treatment of the rat for 19 hrs. with taurine prior to donor nephrectomy [30].  Following transplantation, the taurine preconditioned kidney was resistant to injury (apoptosis and necrosis) relative to the untreated kidney.  Thus, taurine treatment improved graft function during recovery.  Nonetheless, both studies examined the impact of taurine supplementation during an early phase of reperfusion (i.e., 6 hrs).  These beneficial effects resemble the cytoprotective activity of taurine noted in other acute studies, which were attributed to osmoregulation, antioxidation, membrane stabilization, conjugation and regulation of [Ca^2+^]_i_[14].  However, the present study focused on the chronic effects of taurine, which are particularly relevant to recovery of the kidney from IR insult.

A major finding of this study is the differential effect of body taurine status on urinary concentrating ability of the ischemic reperfused kidney.  While the initial decline in urine osmolality was similar in the 3 IR groups, the TT/IR group displayed a gradual, partial recovery of urine osmolality, which resembled that of the C/IR group.  By contrast, the TD/IR group displayed an accelerated, more marked recovery of urinary concentrating ability than that of either the C/IR or the TT/IR group.  Interestingly, the recovery of urine osmolality in the taurine deficient condition is more prominent than has been shown for other maneuvers (e.g., erythropoietin) [31].  A likely contributing factor to the urinary concentrating ability of the post-ischemic kidney relates to dysregulation of renal tubule solute transporters and water channels (i.e., aquaporins) [31,32].  Indeed, expression of aquaporins 2 and 3 in the collecting ducts, as well as expression of aquaporin 1 in the proximal tubules, are significantly reduced in acute renal failure occurring secondary to the IR insult [31,32].  Thus, it is plausible that taurine deficiency modulates the expression of aquaporins (and/or solute transporters), thereby contributing to a more robust recovery of the urinary concentrating ability.  In support of this notion, we now show that the increased baseline urine osmolality is indeed associated with an increase in the renal expression of AQP2, a process that is regulated by arginine vasopressin (AVP) and contributes to the increase in water permeability of the collecting ducts [24].  Collectively, these observations, along with the finding that taurine deficiency increases plasma AVP levels [18], provide compelling evidence for the working hypothesis that taurine deficiency modulates the urinary concentrating mechanism through effects mediated by the release and subsequent renal actions of AVP.  Interestingly, however, taurine supplementation does not significantly affect plasma AVP, urine osmolality [18] or renal aquaporin2 expression.

Another striking, albeit unexpected, difference between the taurine-treated and taurine deficient rats relates to recovery of renal structure from the IR insult. While TD/IR kidneys displayed features resembling sham controls, kidneys of TT/IR rats showed cystic dilatation of renal tubules.  Immunostaining studies using antibodies against renal AQP 1 and 2 suggest a proximal tubule origin for the markedly dilated tubules in the kidneys of TT/IR group; while AQP2 is localized to the distal portions of the nephron, AQP1 is localized to the straight proximal tubules and the early segments of the descending loop of Henle [24].  Thus, it appears that taurine treatment disrupts the normal processes that are involved in the regeneration of the proximal tubules by altering the phenotype of the proximal tubular cells.  These cells, which line the markedly dilated tubules, appear flattened and generally lack brush borders.

Cell volume regulation is critical to both cell proliferation and apoptosis, processes that determine the outcome of an IR insult to the kidney [33,34].  While cell proliferation requires an increase in cell volume, cell shrinkage is a hallmark feature of apoptosis.  Nonetheless, appropriate changes in cell volume under any given condition are dependent on the participation of cell volume regulatory mechanisms [33-36,14].  Taurine is a very important organic osmolyte in mammalian cells, including those of the kidney.  It is a major contributor to regulatory volume processes, such as the regulatory volume decrease and increase, which modulate cell volume and cell membrane stress following exposure of the cell to hypoosmotic and hyperosmotic milieu, respectively [14].  These cellular regulatory volume changes are achieved by ion channels (e.g., Na^+^, Cl^-^) and transporters, including the taurine cotransporter (stoichiometry of 2Na^+^, 1Cl^-^; 1 taurine) [35,14].  Similarly, diverse apoptotic stimuli are known to activate Cl^-^ channels and promote organic osmolyte release (e.g., taurine) [33,34].  Taurine is intimately involved in regulation of these events and the status of the cell in relation to its organic osmolyte content can be of consequence for both cell proliferation and apoptosis.   This is reflected from the results of this study which show differential effects of taurine deficiency and treatment on cell proliferation and apoptosis.  The TD/IR group displayed more proliferative foci but reduced apoptosis while the TT/IR group showed greater apoptosis but reduced proliferative index.  A simple explanation may relate to exposure of renal tubule cells to hyperosmolar urine in the TD/IR than hyposomolar urine in the TT/IR group thereby evoking the regulatory volume increase vs. regulatory volume decrease processes, respectively.  Consequently, the evoked regulatory volume increase should favor cell swelling and proliferation in the TD/IR group while the regulatory volume decrease in the TT/IR should favor cell shrinkage and apoptosis.  Importantly, however, assessment of cell proliferation and apoptosis were carried out 11 days after initiation of IR insult.  Thus future assessment of the influence of body taurine status on temporal changes in cell proliferation and apoptosis in the kidney which is recovering from an IR insult in relation to cell volume regulatory mechanisms is warranted.

Changes in intracellular taurine content not only influence cell proliferation and apoptosis, but also the cellular composition of other osmolytes.  This is understandable since the same transcription factor (i.e., TonEBP/OREBP) regulates transporters and enzymes that participate in cellular accumulation of organic osmolytes [37].  However, if loss of one organic osmolyte was adequately compensated for by another organic osmolyte, one would likely not expect to detect alterations in function/structure of the organ (e.g., the kidney) that relies so heavily on organic osmolytes to cope with hypertonicity of the medullary interstitium [14,20,21].  Yet the present study demonstrates that taurine depletion seemingly benefits the IR kidney.  It is noteworthy that taurine depletion also benefits the IR myocardium [38].  The cardioprotective effect of taurine deficiency may relate, in part, to osmotic preconditioning and activation of prosurvival pathways [39] but their relevance to recovery of the TD/IR kidney remains to be established.

Taurine is known to modulate/regulate inflammatory and immune-mediated processes, aspects that are of particular relevance to the IR kidney.  For example, taurine reacts with hypochlorous acid to form taurine chloramines, a reaction catalyzed by neutrophil myeloperoxidase [40,41].  Taurine chloramine is less toxic and more stable than hypochlorous acid and has been shown to down-regulate the production of pro-inflammatory mediators in leukocytes obtained from human and rodents.  This is achieved, in part, by inhibition of nuclear factor-κB, a potent signal transducer of inflammatory cytokines [40].  Thus, the lack of a beneficial effect of taurine treatment on the function of the ischemic reperfused kidney is inconsistent with a major role for taurine chloramines in the TT/IR kidney.  In fact, the propensity for renal tubules to resemble cyst formations suggests that taurine treatment adversely affects the outcome of the IR insult.  On the other hand, taurine deficiency improved renal structure and function post-insult.  The reasons for these seemingly paradoxical observations are not apparent from this study.  However, it is noteworthy that a number of reports indicate that interruption of inflammatory and immune-mediated processes is beneficial for the recovery of the kidney from an ischemic insult [42,43].  Indeed, the beneficial effect of preconditioning is attributed to the decreased capacity of immune cells to cause tissue injury (43).  In addition, administration of anti-CD44 to mice reduces the influx of neutrophils into the postischemic tissue which is associated with the preservation of renal function [42].  Interestingly, the TT/IR group showed more marked inflammatory cell infiltrates in renal tissue one day after induction of IR injury compared to the TD/IR and C/IR which showed moderate and mild inflammatory infiltrates, respectively.  Thus further studies should explore taurine modulation of inflammatory responses in the setting of renal ischemia reperfusion injury.

## Conclusions

Taurine treatment and taurine deficiency exert differential effects on the outcome of a renal IR insult.  While taurine deficiency seeming benefits the IR kidney, taurine treatment does not affect functional recovery although examination of renal architecture reveals dilated tubules throughout the renal parenchyma.  Given multiple actions of taurine [14,15,39,40], its deficiency is not a desirable clinical objective.  Rather, unraveling of mechanisms of taurine modulation of the IR kidney should enhance our understanding of the role of this ubiquitous but underappreciated renal osmolyte, aspects of potential clinical relevance.

## Abbreviations	

AVP: Arginine vasopressin; AQP: Aquaporin; C: Control; GFR: Glomerular filtration rate; H & E: Hematoxylin-eosin; IR: Ischemia reperfusion; PCNA: Proliferating cell nuclear antigen; SDS-PAGE: Sodium dodecyl sulfate polyacrylamide gel electrophoresis; TD: Taurine deficient; TT: Taurine-treated; TUNEL: Terminal deoxynucleotidyl transferase dUTP nick end labeling.

## Competing interests

The authors declare that they have no competing interests.

## Authors’ contributions

MSM is responsible for the design and overall conduct of the study and preparation of the manuscript.  RA carried out histopathological examination of tissue samples.  CP, HW and JYL conducted the research (80%, 10% and 10%, respectively).  SWS provided valuable comments on the manuscript.
